# Development of Novel Electrospun Fibers Based on Cyclic Olefin Polymer

**DOI:** 10.3390/nano13172412

**Published:** 2023-08-25

**Authors:** Malihe Sabzekar, Mahdi Pourafshari Chenar, Mohamed Khayet, Carmen García-Payo, Seyed Mohammadmahdi Mortazavi, Morteza Golmohammadi

**Affiliations:** 1 Department of Chemical Engineering, Faculty of Engineering, Ferdowsi University of Mashhad, Mashhad 9177948944, Iran; malihe.sabzekar@gmail.com; 2 Department of Structure of Matter, Thermal Physics and Electronics, Faculty of Physics, University Complutense of Madrid, Avda. Complutense s/n, 28040 Madrid, Spain; mcgpayo@ucm.es; 3 Polymerization Engineering Department, Iran Polymer and Petrochemical Institute (IPPI), Tehran 14965/115, Iran; m.mortazavi@ippi.ac.ir; 4 Department of Chemical Engineering, Birjand University of Technology, Birjand 9719866981, Iran; golmohammadi@birjandut.ac.ir

**Keywords:** electrospinning, cyclic olefin polymer, fiber, solvent, post-treatment

## Abstract

For the first time, a systematic study to investigate the electrospinnability of cyclic olefin polymer (COP) was performed. Different solvents and mixtures were tested together with different electrospinning parameters and post-treatment types to prepare bead-free fibers without defects. These were successfully obtained using a chloroform/chlorobenzene (40/60 wt.%) solvent mixture with a 15 wt.% COP polymer, a 1 mL/h polymer solution flow rate, a 15 cm distance between the needle and collector, and a 12 kV electric voltage. COP fibers were in the micron range and the hot-press post-treatment (5 MPa, 5 min and 120 °C) induced an integrated fibrous structure along with more junctions between fibers, reducing the mean and maximum inter-fiber space. When the temperature of the press post-treatment was increased (from 25 °C to 120 °C), better strength and less elongation at break of COP fibers were achieved. However, when applying a temperature above the COP glass temperature (*T_g_* = 138 °C) the fibers coalesced, showing a mechanical behavior similar to a plastic film and a low elongation at break with a high strength. The addition of a high dielectric constant non-solvent, N,N-dimethylacetamide (DMAc), resulted in a considerable reduction in the COP fiber diameter. Based on the cloud point approach, it was found that the use of DMAc and the solvent chloroform or chlorobenzene improved the electrospinnability of COP polymer solution.

## 1. Introduction

Electrospinning has recently gained a surge of interest as a potential technique for developing ultrafine nanofibrous structures due to its simplicity and versatility. Polymeric fibrous structures with fiber diameters ranging from micro- to nano-size exhibit promising characteristics such as a large surface-area-to-volume ratio, high porosity (i.e., void volume fraction), low density, superior mechanical properties, and good flexibility for surface functionalities [1,2]. These advantages make this type of structure an ideal candidate for many technological applications (e.g., tissue scaffolds, energy storage, environmental filtration, catalysts, protective textiles, etc.) [3].

It is well known that submicron-range fibers can be electrospun using strong electrostatic forces which overcome the surface tension of the considered viscoelastic polymer solution, inducing the necessary Taylor cone at the needle tip and producing the electrically charged polymer jet that travels to the collector while it dries relatively through evaporation of the solvent [4]. The properties of electrospun fibers, such as their diameter and morphological structure, void volume fraction, and inter-fiber space size distribution, can be controlled by changing the involved processing parameters (i.e., applied voltage, gap distance, polymer solution flow rate, dimension of the metallic needle, etc.), polymer solution parameters (i.e., polymer concentration, solvent type, electrical conductivity, viscosity, surface tension, etc.), and ambient parameters (i.e., temperature, humidity, and air flow velocity). The success of an electrospinning process is governed by a delicate balance among all the above-mentioned parameters [5]. It is worth mentioning that optimization of electrospinning parameters is still largely carried out on a trial-and-error basis due to the lack of a complete understanding of a complex, variable interplay [6].

Electrospinning is beneficially capable of forming fibers from a wide variety of materials. However, not all materials can be used, considering that a liquid solution must be prepared first and then subjected to phase inversion from liquid to solid. In this sense, various types of polymeric and biopolymeric materials have been considered [7,8,9,10,11]. Moreover, rubber/thermoplastic nanofibers are an innovative category of electrospun material [12,13]. One of the most critical aspects in the development of electrospun polymeric fibers is the appropriate selection of the starting polymer or copolymer [2].

**Figure 1:**
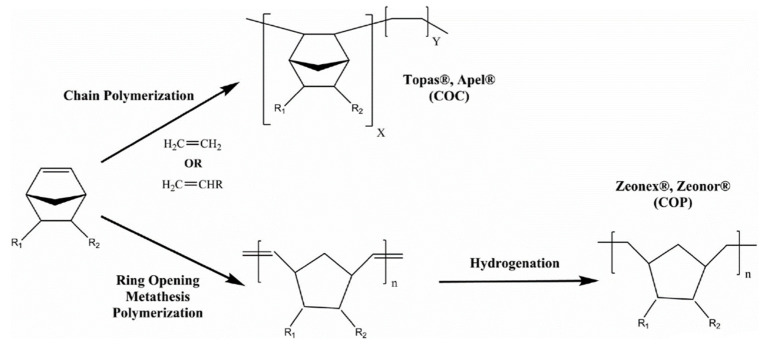
Synthesis of cyclic olefin copolymers (COC) and cyclic olefin polymers (COP) from norbornene [14].

Cyclic olefin polymer (COP) and copolymer (COC) are engineering thermoplastics derived from the ring-shaped norbornene molecule, which can be seen in Figure 1. These are amorphous and inexpensive polyolefins with interesting properties including a high glass transition temperature (*T_g_*: 80–180 °C), good mechanical properties, low moisture absorption, low density, excellent strength and hardness, little shrinkage, good electrical properties, low thermal conductivity, very good melt processability, good biocompatibility (i.e., very good blood compatibility), and high chemical resistance to acids, alkalis, and polar organic solvents [14]. COP is closely related to cyclic olefin copolymer (COC) with some differences including the synthesis process, *T_g_* value, moisture absorption, and cost, among others. As a result of the above-mentioned outstanding properties, COP and COC are attractive in different fields including packaging, optics, and laser technology, microfluids and microdevices [15], membrane technology [16,17], medical equipment, and drug delivery systems [18]. In our previous studies, the first porous COP films were designed and used as membranes for desalination and CO_2_ capture [16,17]. 

To the best of our knowledge, no manuscript about the production of COP electrospun fiber has been published yet. Therefore, the aim of our study is the investigation for the first time of the electrospinnability of COP. The term electrospinnability here refers to the qualification of the polymer solutions to be electrospun for the development of bead-free, homogenous micro-, submicro- or nanofibers [19]. As stated earlier, the successful production of micro/nanofibers by the electrospinning technique requires a sharp-sighted investigation of processing, solution, and environmental parameters. A systematic study for the selection of an appropriate solvent or solvent mixture, polymer concentration, electrospinning parameters, and post-treatment was carried out to develop and evaluate the electrospun COP fibers. The final prepared fibers were evaluated based on their morphological structure, diameters, inter-fiber space, and mechanical properties. 

## 2. Materials and Methods

### 2.1. Materials

Cyclic olefin polymer, Zeonex ^®^ 480R (density: 1.01 g/cm^3^, Melt Flow Index (MFI): 21 g/10 min obtained under a load of 2.16 kg at 280 °C) was supplied by Zeon Europe GmbH (Düsseldorf, Germany). POREFIL^®^ with a surface tension 16 mN/m was employed as a wetting liquid for the porometry measurements.

Other used chemicals in this study were the solvents chloroform (CF, purity, 99–99.4% (GC)), 1,2,4-trichlorobenzene (TCB, anhydrous, ≥99%), chlorobenzene (CB, Synthesis grade, ≥99%), and toluene (T, purity, ≥99.9 (GC)) obtained from Merck. Some properties of interest for these solvents (e.g., density, boiling point, solubility parameter, dipole moment, and dielectric constant) are summarized in Table 1. N,N-dimethylacetamide (DMAc, Synthesis grade, ≥99%) used in this study as non-solvent was also purchased from Merck.

### 2.2. Preparation of COP Fibers

For the preparation of the polymer solutions with different COP concentrations (10, 15, and 20 wt.%), an appropriate amount of COP was dissolved first in different solvents (CF, T, TCB, and CB). The polymer solution was placed in an orbital shaker at 40 °C and 100 rpm until a homogeneous dope solution was achieved. Once the COP solution was prepared, the electrospinning technique was used for the investigation of the electrospinnability of COP. All electrospinning tests were performed at room temperature (23 °C) and 38–41% relative humidity. The electrospinning set-up has been described in detail elsewhere [22]. Briefly, a glass syringe (50 mL, Nikepal, Sacramento, USA) was filled with the polymer solution and an syringe pump (KDS-200, KDS Scientific Inc., Holliston, USA) was used to control the dope solution flow rate. A metallic Hamilton needle having 0.6/0.9 mm as its internal/external diameter and a grounded copper collector covered with aluminum foil were employed as electrodes. An electric voltage was supplied with a DC voltage supply in the kV range (Iseg; model T1CP 300 304P; Iseg Spezialelektronik GmbH, Radeberg, Germany).

For the post-treatment step of the prepared COP fibers, both cold- and hot-pressing were utilized using an electrically heated hydraulic press COLLIN, Maitenbeth, Germany) at different temperatures, pressures, and times. Given that there has been no previous study on COP electrospinning, the involved electrospinning parameters including the polymer solution flow rate, electric voltage, and gap distance were varied to try and find out how to produce continuous and bead-free fiber formation. Finally, the optimum electrospun mats were subjected to a post-treatment. 

### 2.3. Characterization

**Figure 2:**
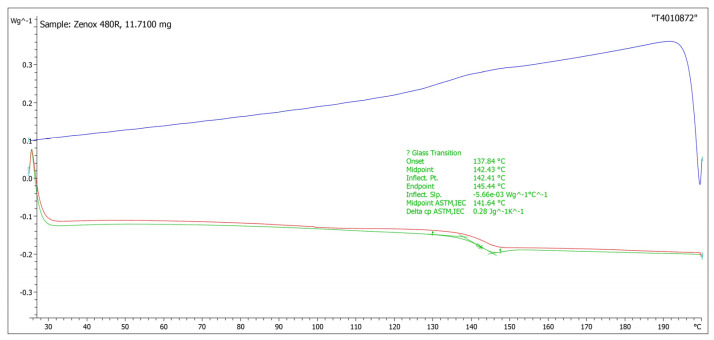
DSC curve of COP (Zeonex 480R).

The differential scanning calorimetry (DSC) thermogram of COP was recorded using a Mettler-Toledo DSC822 instrument (Metler-Toledo, LLC, Columbus, USA) at a heating rate of 10 °C/min within a temperature range of 30–190 °C. The obtained *T_g_* value of the used COP was found to be ~138 °C as shown in Figure 2.

The scanning electron micrographs (SEMs) of the prepared fibers were examined using a LEO 1450 VP SEM, (Zeiss, Oberkochen, Germany) operating at a voltage of 20 kV. The samples were first sputter-coated with a thin gold layer of approximately 5 nm using a rotary-pumped sputter coater (Q150R ES, Quorum Technologies, East Sussex, UK) during 60 s under 20 mA. The diameters of the fibers were determined by analyzing the SEM images of each sample using Image J software (https://www.imagej.nih.gov). For each COP sample, the diameters of at least 50 fibers per image were measured. The mean arithmetic diameter together with its corresponding standard deviation was reported.

The thickness (*δ*) of the COP fibrous mats prepared during the same electrospinning time and different post-treatment conditions was measured using a micrometer equipped with a probe (model 1724-502 series, Helios-Preisser Instruments, Gammertingen, Germany) carrying out at least 30 measurements at different spots. Finally, the mean value of the thickness together with its corresponding standard deviation was reported.

The static water contact angle (*θ*) was measured at room temperature using a computerized optical system CAM100, equipped with a CCD camera, frame grabber, and image analysis software CAM 200 (KSV Instruments Ltd., Monroe, LA, USA). More information can be found elsewhere in [23]. The volume of the droplet of distilled water (~12–14 µL) was controlled by a Hamilton stainless steel needle. Five images were recorded during 4 s for each droplet and at least 10 drops were considered for each sample to determine the average *θ* value together with its standard deviation.

The maximum, mean, and minimum inter-fiber space sizes of the samples were determined using the gas–liquid displacement porometer POROLUX™ 100 (Porometer, Eke, Belgium). After wetting the samples using POREFIL^®^, the S-shaped wet curve was obtained by plotting the air flow rate as a function of the applied hydrostatic pressure difference (0–0.7 MPa) at room temperature (~23 °C). Subsequently, the air flow rate was measured through the dry sample at different hydrostatic pressures to obtain the dry curve. The mentioned parameters were calculated from the obtained cumulative filter flow (*CFF*) and the differential filter flow (*DFF*) curves. At least three tests were performed for each COP fibrous sample. More details can be found elsewhere in [24].

The mechanical properties (tensile strength, elongation at break, and Young’s modulus) of the electrospun COP fibers were investigated according to ASTM D 882 specifications using a universal tensile tester (SANTAM STM20, SANTAM Engineering Design Co. Ltd., Tehran, Iran) equipped with a 6 N load at room temperature, and at a crosshead speed of 5 mm/min on rectangular membrane strips with dimensions of 50 mm × 10 mm. The thickness of each COP fibrous sample was measured and considered in the calculation of the cross-sectional area to determine the tensile strength. Five samples were considered and the average values together with their standard deviations were calculated.

## 3. Results

### 3.1. Effect of Solvent

The selection of an appropriate solvent is a critical key factor affecting the feasibility of electrospinning [25]. The physical properties of solvents have significant impacts on the viscosity, surface tension, and vapor pressure of the polymer solution. It is well known that the Hansen solubility parameter is an effective tool for the investigation of the interaction between the polymer and the solvent [26]. A good solvent favors polymer−solvent interaction, as the polymer chains swell and expand to maximize intermolecular interaction. On the other hand, poor solubility favors polymer−polymer self-interaction, promoting the contraction of polymer chains [27]. Therefore, the selection of a good solvent is a prerequisite for a successful electrospinning.

Another important characteristic of the solvent affecting electrospinning is the electrical conductivity of the polymer solution, which depends on the dielectric constant of solvents. Although there are various solvents to dissolve COP, the chemical resistance of COP to polar solvents, including N-methyl-2-pyrrolidone (NMP) and dimethylformamide (DMF) as recognized electrospinning solvents, creates important restrictions. It is worth quoting that a high electrical conductivity of a polymer solution, which reflects a high charge density of the formed electrified jet, results in a greater level of jet elongation by an electrical force and finally produces smaller fibers [28].

Another crucial role of the solvent in electrospinning is to allow the formation of the electrified polymeric jet at the needle tip, the transport of the formed fiber to the collector, and the final liquid/solid phase inversion through its vaporization [29]. Therefore, for the selection of an appropriate solvent for COP electrospinning, the combination of different properties including the solvent’s affinity to the polymer, the vapor pressure (i.e., boiling point), and the dielectric constant are the most influential parameters. For this purpose, 15 wt.% of COP solution was prepared with different solvents and mixtures of solvents. The best solvents and mixtures of solvents were determined according to visual observation and/or SEM analysis of the obtained morphological structure. Table 2 summarizes the solvents and mixtures of solvents used together with the corresponding visual observations of the obtained structures. For each solvent or mixtures of solvents we tried to obtain fibers by changing the electrospinning parameters including the electric voltage (10–15 kV), the gap distance (10, 15 cm), and the polymer solution flow rate (0.5, 1, and 1.5 mL/h). 

As the solvent CF has a good affinity toward COP based on their solubility parameters (Table 1), it can be considered a good solvent. However, the electrospinning of this polymer solution was not possible due to the needle obstruction, which may be attributed partly to the low boiling point of the CF (~61 °C, Table 1). The COP polymer solution in a single solvent of high solubility such as T or TCB could not be electrospun into fibers, resulting also in the formation of a lot of droplets on the collector. These solvents (T, TCB) have greater boiling points than CF (~110 and 214 °C, respectively) so consequently the needle was not clogged. However, the low dielectric constant of these solvents (T, TCB) resulted in a low electrical conductivity of the polymer solution and consequently no fibers could be formed. It is worth mentioning that T and TCB have almost the same dielectric constants with some differences in the dipole moment, which reflects the polarity of the molecules. A higher dielectric constant and dipole moment of solvents tend to induce a higher electrical susceptibility of the solutions when subjected to an electrostatic field [25]. The qualitative observations of Jarusuwannapoom et al. [30] suggest that the high values of both the dipole moment and the dielectric constant of a given solvent are the most important factors determining the electrospinnability of a polystyrene solution. Other studies [27,31] also claim that the mobility of the electrical charges in a liquid is promoted by an increase in the electrical conductivity, resulting in the formation of thin, uniform, and bead-free fibers. Luo et al. [32], who studied the effect of the solvent dielectric constant on the electrospinning of polycaprolactone solutions, reported that a solvent with a low dielectric constant produced fibers that were micrometers in diameter or electrosprayed. In this case, the selected solvents had similar functional groups and were quite comparable in other physical properties affecting electrospinning. Therefore, the solvent with a high dielectric constant, which indicates the ability of a material to become electrically polarized and store electrical energy, is a strong supporter of an applied electrostatic field. In our case, the electrospinning of the COP solution using CF/T and CF/TCB mixtures of different weights was not possible (Table 2, visual observation). It is worth noting that these mentioned mixtures were used to solve the problem of the low boiling point of CF and/or low dielectric constant of T and TCB. In the next trial, the solvent CB, which has a lower affinity to COP (Table 1, solubility parameters), a higher boiling point, and a higher dielectric constant compared to other solvents was used. The fibers were formed but the collector was wetted to some level by the solvent CB due to its high boiling point. Although this solvent has less affinity to COP compared to CF and T (based on Hansen solubility parameters summarized in Table 1), it showed better electrospinnability than the other solvents. Luo et al. [6] also reported that a partial solubility solvent could result in better electrospinning than a high solubility solvent. The addition of 20 wt.% of CF to CB had no significant effect on the collector wetting by the used solvent, which may have caused melting of the formed fibers. A higher amount of CF in this mixture (50 and 60 wt.%) caused clogging of the needle. However, the addition of 40 wt.% CF to CB resulted in continuous COP electrospinning fibers. Therefore, we fixed the CF/CB mixture to the concentration 40/60 as a good solvent system for a successful COP electrospinning fiber. 

### 3.2. Effect of Electrospinning Parameter

The general effect of processing parameters, including the applied voltage, the flow rate of the polymer solution, and the needle to collector distance, were investigated and reported in the literature [8,33,34]. For instance, the applied electric voltage interacting with the distance between the needle tip and the collector may result in a different morphological structure of the electrospun fibers [35]. The said electrospinning distance should be optimized considering that too short a distance may cause wetting of the collector by the solvent, which may cause melting of the formed fibers on the collector, whereas too long a distance may form discontinuous fibers [36]. In addition, the polymer solution flow rate should also be optimized to induce the necessary jet formation [1]. 

**Figure 3:**
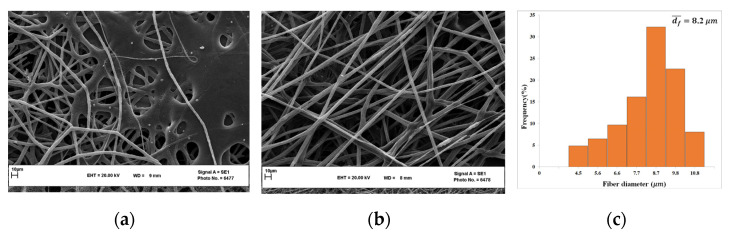
SEM images of the COP fibers prepared with a polymer flow rate of 1.5 mL/h (**a**) and 1 mL/h (**b**) together with their fiber diameter distribution (**c**). (COP concentration: 15 wt.%; solvent mixture: 40/60 CF/CB; gap distance: 15 cm; electric voltage: 12 kV).

The electrospinning of the polymer solution with 15 wt.% COP and 40/60 solvent CF/CB mixture was investigated under different processing parameters. The applied voltage was varied between 10 and 15 kV. As was mentioned previously, the processing parameters were changed in order to guarantee continuous fiber formation. For this reason, the electric voltage was increased from the first value corresponding to the COP polymer jet formation at the needle tip until a maximum value at which a single jet was produced without clogging the metallic needle during electrospinning. Continuous and straight COP fibers with a bead-free structure could be prepared with an electric voltage of 12 kV (Figure 3b). Below this value, the COP fibers showed an unstable electrospinning behavior being formed out of the collector. Multiple jets were formed when the voltage was gradually increased above 12 kV, preventing the preparation of continuous electrospun fibers.

As can be seen in Figure 3c, the diameter of the prepared COP fibers was found to be in the micron range. The increase in electric voltage had no significant effect on fiber diameter. It is worth quoting that different statements have been claimed in the literature related to the change of the fiber diameter with the applied voltage [28,37,38]. It has been reported that the fiber diameter decreases with an increase in electric voltage due to the higher stretching of the fibers exerted by the greater electrostatic forces [37]. In other studies, it has been stated that the fiber diameter increases with an increase in electric voltage due to the faster travel of the charged polymeric jet to the collector and reduced time for solvent evaporation [38]. It has even been reported in some studies that no significant effect of electric voltage on fiber diameter could be detected [28]. These different statements could be attributed partly to the different solvents used. 

The polymer solution flow rate also affects the fiber diameter. In this study, the flow rate was varied as 0.5, 1.0, and 1.5 mL/h. Although a low flow rate is more favorable for electrospinning, 0.5 mL/h was too slow, causing the needle obstruction. On the other hand, when applying a high flow rate, 1.5 mL/h, the fibers coalesced with each other and melted easily, given that there was insufficient time for their drying when reaching the collector plate (i.e., collector wetting with solvent) and due to the high solvent content (Figure 3a) [39]. In this regard, the COP flow rate 1 mL/h was considered as the optimum value, resulting in fine fibers. 

A suitable distance between the needle tip and the collector is necessary for successful electrospinning. This should be long enough for fiber stretching and solvent evaporation, and short enough to prevent any possible discontinuous fiber formation [40,41]. In this case, two distances, 10 cm and 15 cm, were investigated. For the 10 cm gap distance, it was not possible to produce a defect-free COP fibrous structure because of the fusion of fibers similar to the SEM image shown in Figure 3a. When this distance was increased to 15 cm, the COP fibers could be collected without any problem, indicating an adequate distance for complete evaporation of the solvent on the collector. 

Finally, we concluded that the best electrospinning parameters for a successful COP electrospinning fiber are 12 kV electric voltage, 1 mL/h polymer solution flow rate, and 15 cm gap distance. Under these conditions, it was possible to prepare a bead-free COP fibrous structure with continuous electrospun fiber formation. 

A summary of different electrospinning conditions along with the corresponding visual observations is reported in Table 3. In all electrospinning tests, the COP concentration, 15 wt.%, and solvent mixture, CF/CB (40/60), were maintained the same. 

### 3.3. Effect of Polymer Concentration

The polymer concentration together with the solvent(s) exert a fundamental influence on the electrospinnability of the polymeric solution as both affect its viscosity, electrical conductivity, and surface tension. It has been reported that the polymer concentration should exceed a critical minimum concentration (CMC) in order to achieve a good molecular chain entanglement that prevents beads formation [28]. A polymer concentration below CMC results in electrospraying, while a higher concentration dominates the onset of the transition from electrospraying to electrospinning and consequently the shape of the beads changes from spherical to spindle-shaped and finally to uniform fibers [27,42]. The higher the concentration of the polymer, the more chain entanglement and the less chain mobility can be achieved to stabilize the charged jet, decreasing therefore the disruptions during electrospinning, and consequently favoring the formation of thicker fibers [43]. 

**Figure 4:**
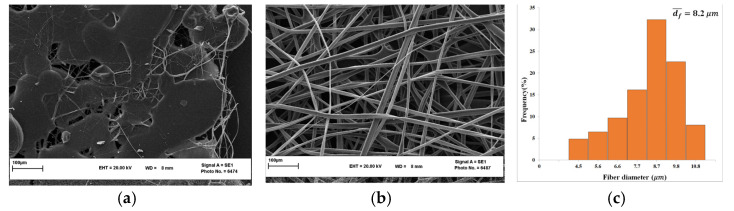
SEM images of COP fibers prepared with different COP concentrations: 20 wt.% (**a**) and 15 wt.% (**b**) together with their fiber diameter distribution (**c**). (Polymer solution flow rate: 1 mL/h; solvent mixture: 40/60 CF/CB; gap distance: 15 cm; electric voltage: 12 kV).

For COP electrospinning with the CF/CB solvent mixture, three different concentrations (10 wt.%, 15 wt.%, and 20 wt.%) were examined to evaluate the effect of COP concentration on the morphological structure of the fibers. A concentration of 10 wt.% COP produced droplets on the collector because it is such a low concentration and insufficient to induce chain entanglements. Han et al. [44] stated that a low polymer concentration leads to a low surface tension of the polymer solution so that the droplets cannot be fully stretched during the ejection. In this study, fibers could be prepared using the higher COP concentration, 20 wt.%, but most of them were finally melted and mixed with many accumulated droplets on the collector (Figure 4a). Instead, smooth micron fibers could be prepared using a 15 wt.% COP concentration (Figure 4b,c). With the increase in the polymer concentration up to 20 wt.%, the mobility of the polymeric chain decreased, preventing chain re-ordering, and eventually the viscosity of the polymer solution was enhanced. This limited the process stability and compromised the continuous flow of the polymer solution [45]. 

### 3.4. Effect of Post-Treatment Conditions

Electrospinning was carried out for 2 h and fiber mats with a cotton-like structure were prepared using a 15 wt.% COP concentration, the CF/CB solvent mixture (40/60), a polymer solution flow rate of 1 mL/h, a needle/collector distance of 15 cm and an electric voltage of 12 kV. To increase the mechanical strength together with the structural integrity of the polymeric electrospun mats, different post-treatments have been adopted [46]. In this study, both cold-press and hot-press post-treatments were applied. 

**Figure 5:**
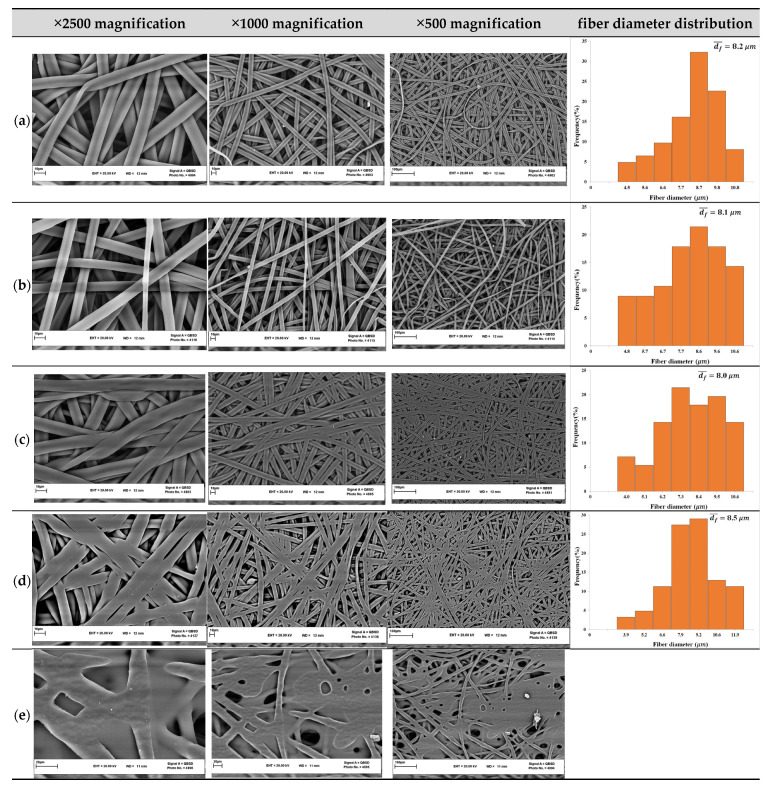
SEM images of COP fibers after the post-treatment step: as spun COP fibrous mat (**a**), with cold-press (10 MPa, 5 min) (**b**), with hot-press (5 MPa, 5 min) at 100 °C (**c**), 120 °C (**d**), and 140 °C (**e**) at different magnifications together with their fiber diameter distribution. (COP concentration: 15 wt.%; solvent mixture: 40/60 CF/CB; polymer solution flow rate: 1 mL/h; electric voltage: 12 kV; gap distance: 15 cm).

The effect of the cold-press post-treatment of the COP fibrous structure was investigated at two different pressures, 5 and 10 MPa, for 5 min. The SEM images of the cold-pressed COP fibers is shown in Figure 5b. Although the appearance of the fibers (top and bottom layers) was improved for both applied pressures, no junction points were formed between fibers. The inner layers of the COP fibrous structure remained cotton-like. 

The hot-press post-treatment was carried out at 5 MPa for 5 min and different temperatures (100, 120, and 140 °C). As stated earlier, the *T_g_* of COP was found to be 138 °C. The SEM images of the resultant fibrous structure are shown in Figure 5c–e. It can be seen that the loose fibrous structure became more compacted, and junctions were formed between fibers under the hot-press post-treatment conditions of 5 MPa, 5 min, and 100 °C (Figure 5c). The cotton-like fibrous structure became more integrated. The increase in temperature to 120 °C caused the fusion of fibers along with more junctions (Figure 5d). However, the enhancement of the temperature to 140 °C, which is slightly higher than the *T_g_* of COP, resulted in many fibers melting (Figure 5e). This rendered many parts of the COP film almost non-porous and transparent. This fibrous mat was not subjected to further characterization. 

The thickness, water contact angle, mean fiber diameter, inter-fiber space (minimum, mean, and maximum) of each of sample were measured and are reported in Table 4. Both post-treated and untreated COP fibrous mats were characterized. As mentioned previously, the untreated COP fibrous mat exhibited a loose cotton-like structure, and the measurement of its water contact angle was impossible. In addition, this sample showed the highest thickness, fiber diameter, and inter-fiber space. When this COP fibrous mat was subjected to cold-press post-treatment, the thickness was decreased considerably, from 140 µm to 92 µm (34.3%, Table 4). The water contact angle of COP fibers (130°) was found to be greater than that of COP phase inversion flat sheets (111°) [16]. This is due partly to the higher surface roughness of the fibrous structure and the air entrapped between the fibers. Taking into consideration the resulting standard deviation, the fiber diameters did not show any significant change after the cold-press post-treatment. Similarly, the minimum and mean inter-fiber space sizes remained almost unchanged. A very small reduction in the mean inter-fiber space could be detected (less than 7%). On the contrary, with the application of the cold-press post-treatment a significant decrease was registered for the maximum inter-fiber space. This marked reduction could be due to the more compacted fibrous structure formed after the pressing treatment, reducing mainly the maximum inter-fiber space. 

Compared to the cold-press post-treatment, the hot-pressed COP fibrous mat resulted in a greater decrease in the thickness (up to 48.9%) due to the greater compaction and fusion of the electrospun fibers. As a result of this compaction, the surface became smoother and consequently the water contact angle was reduced (from 130° to 125.4°). Upon hot-pressing the COP fibrous mat, the inter-fiber space was reduced more than the observed decrease after cold-pressing due to the favored junctions and contacts between fibers. The fiber diameters were found to be in the micron range and no significant change could be detected by the different post-treatments applied. By increasing the hot-press temperature from 100 °C to 120 °C, a slight increase in the fiber diameter was observed. This might be due to the flattening of the fibers because of the applied pressure at a higher temperature. 

**Figure 6:**
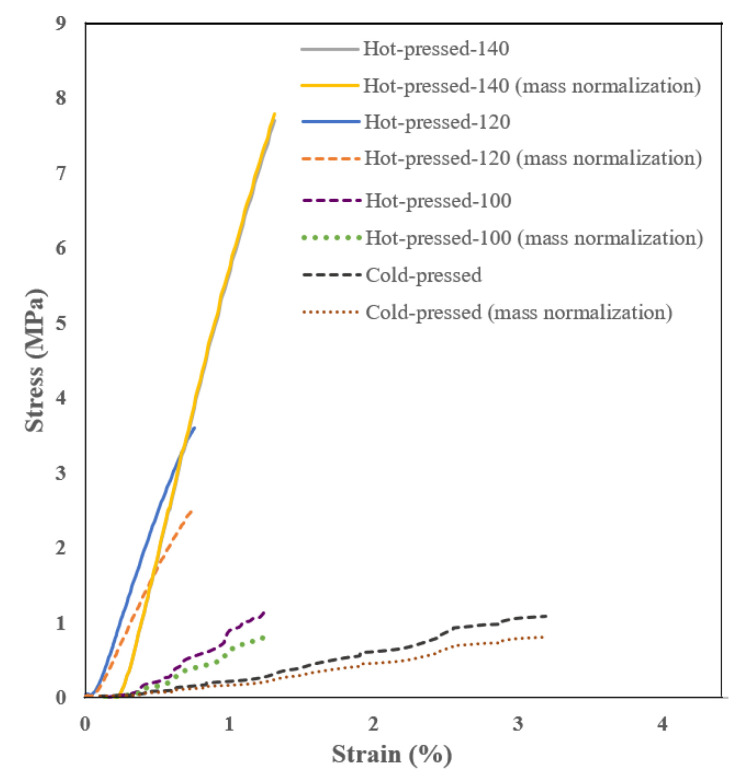
Stress–strain curves by normalizing the load with respect to cross-section and specimen mass based on the procedure reported in [47].

The mechanical properties of COP fibers, including tensile strength, elongation at break, and Young’s modulus, were evaluated and the obtained results are reported in Table 5. Those of the untreated COP fibrous mat were not able to be carried out because of its weak integrity (i.e., loose cotton-like structure). Both the cold-press and hot-press post-treatments improved the tensile strength and Young’s modulus of the COP fibrous mat. The cold-pressed fibrous mat, which showed almost a cotton-like structure, especially at its inner layer, had the lowest tensile strength (1.1 MPa) and Young’s modulus (35.6 MPa). On the contrary, the hot-pressed sample at 140 °C, which was almost changed to a plastic film, exhibited the highest tensile strength (6.8 MPa, Table 5) and Young’s modulus (575 MPa). Consequently, the cold-pressed fibrous mat could be more elongated (70%) compared to the hot-pressed samples. As the Young’s modulus represents the stiffness of a material, it was expected that the COP plastic film (hot-pressed at 140 °C) exhibited the maximum value (575 MPa). It is known that a flexible material has a low Young’s modulus. Therefore, the cold-press COP fibrous mat having a greater elongation at break (70%) showed a low Young’s modulus. As the hot-press temperature was enhanced, the COP fibrous mat showed a more compacted structure with more junctions and coalesced fibers, higher tensile strength, lower elongation at break and consequently a greater Young’s modulus as a result of more stiffness. Maccaferri et al. [47] claimed that the stress obtained by normalizing the load with respect to the cross-area is unreliable in the case of highly porous materials such as fibrous/nanofibrous mats. They proposed instead normalization based on mat grammage and the specimen mass. They concluded that the normalization based on the specimen mass was more reliable. Details of their followed procedure are discussed in detail in [47]. In our case, the data of load-displacement curves were normalized based on the specimen mass and the results are plotted in Figure 6. It is worth quoting that for the COP fibrous structure and the observed decrease in the tensile strength and modulus are due to the mass consideration of samples. In addition, the stress–strain curve of the COP hot-pressed at 140 °C showed no change due to the severe coalescence of the fibers resulting in a plastic film. 

### 3.5. Effect of Non-Solvent

It is well known that polar solvents with a high dielectric constant and dipole moment are good solvents for electrospinning of different polymers [35]. However, COP is miscible in non-polar solvents with an almost low dielectric constant. One of the major problems of such non-polar solvents is their inability to withstand the electrospinning technique. In this study, the best mixture of solvents for COP electrospinning was found to be CF/CB with a concentration of 40/60 as shown in the previous sections. Based on the cloud point approach we investigated the electrospinnability of COP fibers using single solvents (CF and CB) and the non-solvent DMAc because of its high dielectric constant.

It must be pointed out that after adding only a very small amount of DMAc in the COP solution (~0.01 wt.% in the polymer solution) prepared with the solvent mixture CF/CB (40/60), the polymer solution became turbid so that electrospinning was impossible. It was observed that lower amounts of DMAc (<0.01 wt.%) had no significant effect on COP electrospinning. Therefore, DMAc was then added to COP/CF and COP/CB polymer solutions until these became turbid. It was found that the higher DMAc amounts added in the polymer solutions before turbidity were ~0.04 wt.% and 0.06 wt.% for the COP/CF and COP/CB polymer solutions, respectively. In this case, the COP concentration after the addition of DMAc was ~15 wt.% and electrospinning was performed following the same optimum condition applied in the previous Section 3.2 (12 kV electric voltage; 15 cm gap distance; and 1 mL/h polymer solution flow rate). Visual observations of the obtained mats showed that continuous COP electrospinning took place for both dope solutions (COP/CF and COP/CB) containing 0.04 wt.% and 0.06 wt.% DMAc, respectively. It is interesting to note that electrospinning of COP solution prepared with CF or CB alone as a solvent was impossible. However, the addition of the non-solvent DMAc to these solutions allowed fiber formation. 

**Figure 7:**
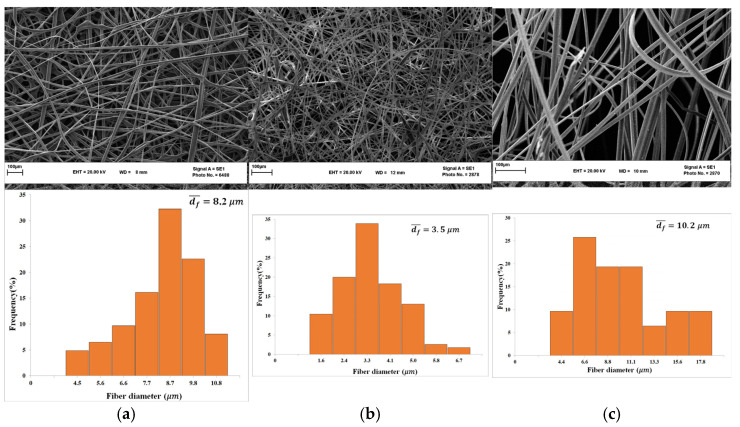
SEM images of COP fibrous mats prepared with the solvent mixture CF/CB 40/60 (**a**), CB/DMAc (**b**), and CF/DMAc (**c**) together with their fiber diameter distributions. (Polymer solution flow rate: 1 mL/h; gap distance: 15 cm, and electric voltage: 12 kV; final concentration of COP after DMAc addition; ~15 wt.%).

The SEM images of the obtained fibrous mats together with the diameter distributions of the electrospun fibers are depicted in Figure 7. The COP/CF/DMAc solution allowed the production of bigger fibers (i.e., average fiber diameter = 10.2 ± 4.1 μm) compared to the COP fibers prepared with the solvent mixture CF/CB (40/60) without DMAc (i.e., average fiber diameter = 8.2 ± 1.9 μm), whereas the electrospinning of COP/CB/DMAc solution resulted in the formation of COP fine fibers with smaller diameters (i.e., average fiber diameter = 3.5 ± 1.2 μm). 

## 4. Conclusions

To the best of our knowledge, for the first time, the electrospinning of cyclic olefin polymer (COP) was performed to investigate its possible electrospinnability. Different solvents (chloroform, CF; toluene, T; 1,2,4-trichlorobenzene, TCB; and chlorobenzene, CB) were tested together with their mixtures to find out the good solvent(s) for COP fiber formation. After the examination of different COP concentrations of the polymer solution (10, 15, and 20 wt.%), different electrospinning parameters such as the electric voltage, the needle-to-collector distance, and the polymer solution flow rate were tested. A bead-free COP fibrous structure was achieved using a CF/CB solvent mixture (40/60) for 15 wt.% of COP and the electrospinning parameters: 12 kV electric voltage, 15 cm gap distance, and 1 mL/h polymer solution flow rate. Since the obtained COP fibrous structure was loose and cotton-like, different post-treatments including cold and hot-press at different temperatures were investigated. The morphological structure analysis revealed that micron-range COP fibers could be prepared. The hot-press post-treatment (5 MPa, 5 min, and 120 °C) improved COP fibrous integrity and compaction, inducing junctions and fusion between fibers. This resulted in a considerable decrease in the fibrous mat thickness together with the maximum and mean inter-fiber space, while the fiber diameter and the small inter-fiber space hardly underwent changes. The post-treatment at a temperature above the COP glass temperature led to a severe melting of most fibers, rendering a large part of the sample transparent, similar to plastic. Compared to other samples, the cold-press fibrous mat exhibited the lowest tensile strength and Young’s modulus but the highest elongation at break. By increasing the hot-press temperature from 100 °C to 120 °C, the COP fibrous mat became more compacted with more junctions and coalesced fibers, with higher tensile strength, lower elongation at break, and consequently a greater Young’s modulus. Based on the cloud point approach, the effect of the non-solvent additive N,N-dimethylacetamide (DMAc) having a high dielectric constant on the electrospinnability of the COP/CF and COP/CB solutions was also investigated. Interestingly, a reduction in the COP fiber diameter by half was observed with the addition of DMAc to the COP/CB solution.

## Figures and Tables

**Table 1:** Properties of the used solvents ^+^ (chloroform, CF; 1,2,4-trichlorobenzene, TCB; chlorobenzene, CB; toluene, T) and the non-solvent N,N-dimethylacetamide (DMAc).

Solvent	Density (g/cm^3^)	Viscosity at 25 °C (mPa.s)	Boiling Point (°C)	Solubility Parameter (MPa ^1/2^)	Dielectric Constant (-)	Dipole Moment (D)
COP *	1.01	-	-	18.3	2.3	-
Chloroform (CF)	1.49	0.54	61	18.9	4.81	3.44
Toluene (T)	0.87	0.56	111	18.2	2.38	0.37
1,2,4- trichlorobenzene (TCB)	1.46	1.93	214	21.3	2.24	1.26
Chlorobenzene (CB)	1.11	0.74	130	19.6	5.62	1.50
N,N-dimethylacetamide (DMAc)	0.94	0.95	166	22.8	37.8	3.72

^+^ Properties of solvents were adopted from [20]. * Molecular weight of COP (480R): 480,000 g/mol was adopted from [21].

**Table 2:** Effect of different solvents and mixtures of solvents on COP electrospinning samples. (COP concentration and electrospinning conditions were kept the same for all samples).

No.	Solvents and Mixtures	Concentration (wt.%)	Visual Observation
1	CF	100	Needle obstruction/non-continuous electrospinning
2	T	100	No fibers/droplets formed on the collector
3	CF/T	90/10	Needle obstruction
4	CF/T	80/20	Droplets formed on the collector/non-continuous electrospinning
5	TCB	100	No fibers/droplets formed on the collector
6	CF/TCB	90/10	Droplets formed on the collector/non-continuous electrospinning
7	CB	100	Fibers/collector wetting by CB solvent and melting of the formed fibers on the collector
8	CF/CB	20/80	No fibers/collector wetting by CF/CB solvents and melting of the formed fibers on the collector
9	CF/CB	40/60	Continuous fiber formation

**Table 3:** Effect of different electrospinning parameters on the formed COP fibers and fibrous structure.

No.		Voltage (kV)	Polymer Flow Rate (mL/h)	Needle-Collector Distance (cm)	Observation
1	Tests to find the appropriate voltage	10	1	15	Fiber formation outside the collector
2		12			Continuous fiber formation on the collector
3		15			Continuous fiber formation on the collector
4		17			Multiple COP polymer jets (non-continuous electrospinning)
5	Tests to find the appropriate flow rate	15	0.5	15	Needle obstruction
6			1		Continuous fiber formation on the collector
7			1.5		Melting of the formed fibers on the wetted collector by the used solvent
8	Tests to find the appropriate needle-to-collector distance	15	1	10	Melting of the formed fibers on the wetted collector by the used solvent
9				15	Continuous fiber formation on the collector

**Table 4:** Thickness (*δ*); water contact angle (*θ*); fiber diameter; and minimum, mean, and maximum inter-fiber space of un-treated and post-treated COP fibrous mats.

Post-Treatment Technique	*δ* (µm)	*θ* (°)	Fiber Diameter (µm)	Minimum Inter-Fiber Space (µm)	Mean Inter-Fiber Space (µm)	Maximum Inter-Fiber Space (µm)
Without post-treatment	140 ± 20	-	8.2 ± 1.5	2.9 ± 2.3	10.0 ± 1.6	50 ± 20
Cold-press	92 ± 3	130.0 ± 2.3	8.1 ± 2.3	2.7 ± 1.4	9.1 ± 2.4	20 ± 4
Hot-press (100 °C)	81 ± 5	126.1 ± 1.5	8.0 ± 2.5	2.2 ± 0.5	7.1 ± 1.2	10 ± 2
Hot-press (120 °C)	80 ± 4	125.4 ± 1.7	8.5 ± 1.4	2.4 ± 0.9	7.3 ± 1.1	8.3 ± 1.4

**Table 5:** Mechanical properties of electrospun COP fibrous mats subjected to different post-treatment conditions.

Sample	Tensile Strength (MPa)	Elongation at Break (%)	Young’s Modulus (MPa)
Cold-press	1.1 ± 0.6	70 ± 12	35.6 ± 4.2
Hot-press (100 °C)	1.4 ± 0.2	30 ± 8	103 ± 10
Hot-press (120 °C)	3.2 ± 0.4	1.5 ± 0.3	427 ± 14
Hot-press (140 °C)	6.8 ± 0.7	0.9 ± 0.5	575 ± 20

## Data Availability

This study did not report any data.
